# Efficacy of a new ready-to-use vaccine against PCV-2d and *Mycoplasma hyopneumoniae* under experimental conditions

**DOI:** 10.17221/6/2025-VETMED

**Published:** 2025-06-30

**Authors:** Roman Krejci, Peter Trampus, Attila Csagola, Tamas Szalai, Nimrod Palmai, Adam Toth, Nora Terenyi, Zoltan Nagy, Nikoletta-Agnes Szeplaki, Gergely Somogyi, Timea Barna, Eniko Rausch, Zoltan Penzes, Maria Szaszko, Zsolt Lorincz

**Affiliations:** ^1^Ceva Sante Animale, Libourne Cedex, France; ^2^Ceva-Phylaxia Zrt., Budapest, Hungary; ^3^TargetEx Biosciences Ltd., Dunakeszi, Hungary

**Keywords:** enzootic pneumonia, PCV-2 disease, vaccination

## Abstract

*Mycoplasma hyopneumoniae* (*M. hyo*) and porcine circovirus type 2 (PCV-2) are major and widespread swine pathogens, both implicated in the porcine respiratory disease complex, which can lead to significant economic losses for pig producers. PCV-2d is currently the most prevalent genotype. Vaccination against these two pathogens helps mitigate their impact on pig health and performance. The use of ready-to-mix or ready-to-use (RTU) vaccines targeting PCV-2a or PCV-2a/b and *M. hyo* is a common practice. This study aimed to evaluate the efficacy of a novel RTU vaccine containing PCV-2d and *M. hyo* antigens under experimental conditions. Several challenge trials were conducted using PCV-2a, PCV-2b, PCV-2d, and *M. hyo* to assess the level of protection conferred against different PCV-2 genotypes and *M. hyo*, as well as to determine the duration of protection. This study demonstrated that the bivalent PCV-2/*M. hyo* vaccine induces both early and long-lasting protection against infections caused by *M. hyo* and PCV-2. In addition, cross-protection against the three major PCV-2 genotypes was confirmed.

Porcine circovirus type 2 (PCV-2) and *Mycoplasma hyopneumoniae* (*M. hyo*) are major swine pathogens with widespread distribution in most pig farms. PCV-2 is currently one of the most important viral pathogens affecting pigs. PCV-2 infection is associated with various disease syndromes, particularly during the grow-finish phase, collectively referred to as porcine circovirus diseases (PCVD) (Segales and Sibila 2022). PCV-2 has also been recognised as a significant contributor to the porcine respiratory disease complex (PRDC). *M. hyo* is the primary aetiological agent of enzootic pneumonia (EP) and is the most important bacterial pathogen involved in PRDC. *M. hyo* infection leads to the degeneration of bronchial cilia, thereby compromising the first line of pulmonary defence. Subsequently, the overactivation of the local immune response leads to the development of bronchopneumonia, characterised by typical lung lesions, such as ventrocranial pulmonary consolidation.

Although PCV-2 and *M. hyo* target different organs and cell types, co-infection with both pathogens results in more severe clinical respiratory disease, reduced weight gain, more extensive lung lesions, and an increased amount and prolonged presence of PCV-2 antigen (Opriessnig et al. 2011). It has been demonstrated that *M. hyo* exacerbates the severity of subsequent PCV-2 infection. Co-infection with both agents leads to higher respiratory scores – particularly in cases involving *M. hyo* and PCV-2d – and poorer growth performance. *M. hyo* has been shown to enhance the replication of PCV-2d more significantly than other PCV-2 genotypes (Oh et al. 2021). Infections with PCV-2 and *M. hyo* also predispose pigs to secondary infections, increasing overall morbidity and adversely affecting performance.

Prevention of PCVD and EP is widely practiced on swine farms through piglet vaccination. The level of maternally derived immunity, which could interfere with vaccine-induced responses, typically declines rapidly for both PCV-2 and *M. hyo*. This decline allows for effective vaccination from three weeks of age, usually around weaning. Simultaneous vaccination against PCV-2 and *M. hyo* is convenient, cost-effective, and piglet-friendly. Although single monovalent and bivalent ready-to-mix (RTM) or ready-to-use (RTU) vaccines are available on the market, questions have arisen, particularly concerning the level of *M. hyo* protection under field conditions.

Epidemiological and phylodynamic studies have revealed shifts in PCV-2 genotypes (Franzo and Segales 2020). PCV-2a was the most prevalent genotype in clinically affected pigs between 1996 and the early 2000s. This was followed by the emergence of PCV-2b, which was associated with more severe clinical outbreaks. Subsequently, a global shift from PCV-2b to PCV-2d occurred, with the latter now frequently – or even exclusively – detected in clinically affected herds. For instance, PCV-2d was identified in 100% of isolates from diseased pigs in Belgium (Wei et al. 2019), 100% in Spain (Sibila et al. 2021), and 60% in Austria (Weissenbacher-Lang et al. 2020). Nevertheless, the detection of other PCV-2 genotypes in vaccinated herds is still not uncommon. When random sampling is performed, PCV-2a and PCV-2b are still found circulating within pig populations.

A new RTU vaccine, Cirbloc M Hyo (Ceva Santé Animale, Libourne, France), combining PCV-2d and *M. hyo* antigens, has been developed and evaluated under experimental conditions.

## MATERIAL AND METHODS

### Vaccine

Cirbloc M Hyo (Ceva) contains a whole-cell concentrate of inactivated *M. hyo* and the ORF2 capsid protein of a PCV-2d “consensus” strain, presented in the form of virus-like particles expressed using the baculovirus expression system. The PCV-2d sequence represents a consensus derived from isolates circulating in China, the EU, and the US. The vaccine is formulated with a proprietary oil-in-water adjuvant specifically developed for this antigen combination. It is intended for the active immunisation of pigs to reduce viraemia, viral load in the lungs and lymphoid tissues, virus shedding due to PCV-2 infection, and the severity of lung lesions caused by *M. hyo* infection.

### Animals

All laboratory (pre-clinical) studies were conducted on 3-week-old piglets, which represent the target species, age, and category. Conventional piglets were sourced from a farm positive for PCV-2 and *M. hyo*, but with a stable, low infection rate. In the *M. hyo* onset of immunity (OOI) and duration of immunity (DOI) studies, 20 and 27 animals were included, respectively. For the PCV-2 OOI and DOI studies, 15 and 20 animals were used, respectively. In each study, the vaccine was administered once, as recommended, via intramuscular injection at a dose of 2 ml.

### Challenge model

The protective efficacy of the vaccine was evaluated through challenge studies, with the validity of each challenge confirmed by the inclusion of a challenge control group. The observation post-challenge period was four weeks for both pathogens.

The efficacy of the *M. hyo* component was demonstrated in accordance with Pharmacopeial requirements (Ph. Eur. 2448), while an in-house challenge model was used to evaluate PCV-2 efficacy. Immunity against EP was tested using the pathogenic *M. hyo* strain L1, administered intratracheally for three consecutive days (10 ml of 1 × 108, 1 × 108, and 5.5 × 108 CCU/animal), either 3 weeks (OOI) or 23 weeks (DOI) post-vaccination. The primary efficacy endpoint was lung lesion score, evaluated according to Ph. Eur. 2448 guidelines. Lung lobes were scored from 0 to 5 based on the percentage of consolidation, with major alterations such as atelectasis and hyperplastic inflammation in the peribronchial area considered typical. The weighted lung scores were calculated using lobe-specific weighting factors described in the Ph. Eur. 2448 monograph. The maximum score was 7 × 5 = 35, and with weighting, the maximum total score per animal was 500.

For PCV-2, challenge strains representing different genotypes (PCV-2a, PCV-2b, and PCV-2d) were used. Pigs were infected intranasally with 3 ml per nostril. All challenge viruses were heterologous to the vaccine strain. The PCV-2a challenge dose was 5.0 log_10_ TCID_50_/piglet, PCV-2b was 4.77 log_10_ TCID_50_/piglet, and PCV-2d was 9.36 log_10_ copies/piglet. OOI studies involved infection with PCV-2a, PCV-2b, or PCV-2d strains two weeks post-vaccination. In the DOI study, pigs were challenged with PCV-2d at 23 weeks post-vaccination using a dose of 6.3 log_10_ copies/piglet. PCV-2 challenge efficacy was assessed by quantifying viral loads using real-time PCR in the serum, tonsils, and mediastinal, mesenteric, and inguinal lymph nodes (l. n.), as well as lung samples collected four weeks post-challenge.

### Statistical analysis

Different types of parameters were analysed using appropriate statistical methods. All statistical analyses were performed using Stata v15, or higher (StataCorp LLC, USA) and R v4.1.2 (R Core Team), including the *tidyverse* and *readxl* packages. A significance level of 0.05 was applied in all cases.

Mean PCV-2 viral loads in lymph nodes, tonsils, and lung samples were compared between groups using the Wilcoxon rank-sum test (Mann–Whitney test) or the *t*-test followed by Welch’s correction. Before statistical testing, data were log-transformed. Mean weighted lung scores were compared between groups using Welch’s *t*-test.

## RESULTS

### Efficacy of Cirbloc M Hyo vaccine against *M. hyo* challenge

The efficacy of the *M. hyo* component of Cirbloc M Hyo was demonstrated at 3 weeks after immunisation of 3-week-old piglets. A statistically significant difference (*P* < 0.05) in lung lesion scores was observed between the groups, with significantly lower scores in the vaccinated group (*P* = 0.000 6; Welch test) (Table 1).

**Table 1:** Summary of the mean weighted lung lesion scores of the groups challenged at 3 weeks post-vaccination (6 weeks of age)

Group	*N*	Mean	SD	Median	Minimum	Maximum
Cirbloc M Hyo	19	23.1	25.4	15.0	0.0	71.0
Control	20	72.4	51.1	80.0	0.0	147.0

The efficacy of the *M. hyo* component of Cirbloc M Hyo was also demonstrated at 23 weeks post-immunisation of 3-week-old piglets. Again, the Welch test indicated that lung lesion scores were significantly lower in the vaccinated group (*P* = 0.001 7) (Table 2).

**Table 2:** Summary of the mean weighted lung lesion scores of the groups challenged at 23 weeks post-vaccination (26 weeks of age)

Group	*N*	Mean	SD	Minimum	Maximum
Cirbloc M Hyo	23	47.3	43.89	0	153
Control	25	86.1	35.54	21	162

### Efficacy of Cirbloc M Hyo vaccine against PCV-2 challenge

#### EFFICACY AGAINST PCV-2A IN PIGS CHALLENGED 2 WEEKS AFTER VACCINATION

All organ samples from vaccinated pigs exhibited significantly lower PCV-2 loads compared to those from control animals.

Vaccination reduced PCV-2 loads by approximately 100- to 1 000-fold (Wilcoxon rank-sum test) (Table 3).

**Table 3:** Geometric mean PCV-2 DNA copy numbers in organ samples (log_10_ copy number/ml)

Groups	*N*	Tonsils	Mediastinal l. n.	Mesenteric l. n.	Inguinal l. n.	Lung
Cirbloc M Hyo	15	4.6	6.2	4.5	5.2	6.1
Control	15	7.5	8.0	7.9	7.3	7.4
Statistical significance		*P* < 0.000 1	*P* = 0.010 4	*P* < 0.000 1	*P* < 0.000 1	*P* = 0.000 1

l. n. = lymph nodes

#### EFFICACY AGAINST PCV-2B IN PIGS CHALLENGED 2 WEEKS AFTER VACCINATION

All organ samples in the vaccinated group showed significantly lower PCV-2 loads compared to those in control animals. PCV-2 loads were markedly reduced in the vaccinated pigs (Wilcoxon rank-sum test) (Table 4).

**Table 4:** Geometric mean PCV-2 DNA copy numbers in organ samples (log_10_ copy number/ml)

Groups	*N*	Tonsils	Mediastinal l. n.	Mesenteric l. n.	Inguinal l. n.	Lung
Cirbloc M Hyo	15	6.6	7.6	6.8	6.6	6.9
Control	15	9.9	9.9	10.0	9.1	8.5
Statistical significance		*P* = 0.000 0	*P* = 0.000 1	*P* = 0.000 0	*P* = 0.000 0	*P* = 0.000 0

l. n. = lymph nodes

#### EFFICACY AGAINST PCV-2D IN PIGS CHALLENGED 2 WEEKS AFTER VACCINATION

All organ samples from the vaccinated group exhibited significantly lower PCV-2 loads than those from control animals. Vaccination with Cirbloc M Hyo reduced PCV-2 load by up to approximately 10^5^-fold (Table 5).

**Table 5:** Geometric mean PCV-2 DNA copy numbers in organ samples (log_10_ DNA copy number/ml)

Group	*N*	Tonsils	Mediastinal l. n.	Mesenteric l. n.	Inguinal l. n.	Lung
Cirbloc M Hyo	12	4.8	4.1	5.1	5.0	5.8
Control	14	10.1	8.4	9.6	9.4	8.5
Statistical significance		*P* = 0.000 0	*P* = 0.001 5	*P* = 0.000 0	*P* = 0.000 0	*P* = 0.000 0

l. n. = lymph nodes

#### EFFICACY AGAINST PCV-2D AT 23 WEEKS POST-VACCINATION

**Figure 1:**
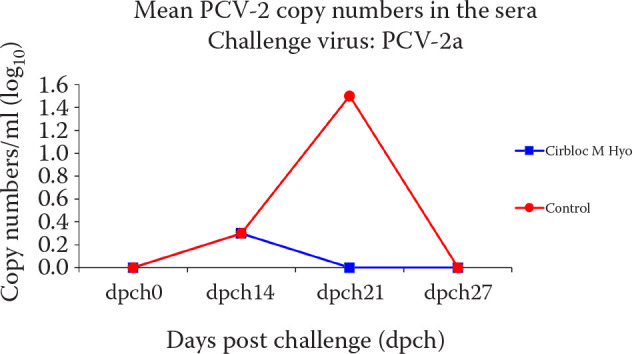
Geometric mean PCV-2a DNA copy numbers in the sera (log_10_ DNA copy number/ml) The AUC of the viraemia was not significantly different between the groups (*P* = 0.125 2, Wilcoxon rank-sum test). However, the level of viraemia was lower in the vaccinated group AUC = area under the curve

**Figure 2:**
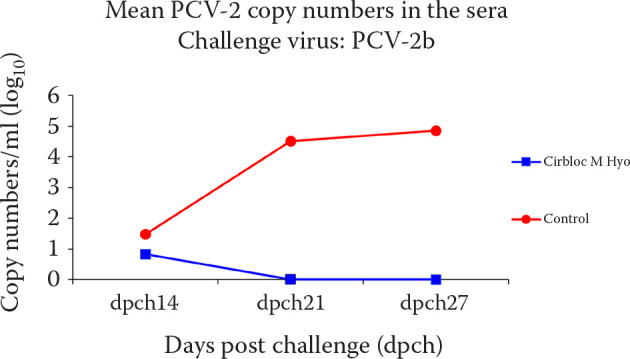
Geometric mean PCV-2b DNA copy numbers in the sera (log_10_ DNA copy number/ml) The AUC of the viraemia was significantly different between the groups (*P* = 0.000 0, Wilcoxon rank-sum test) AUC = area under the curve

**Figure 3:**
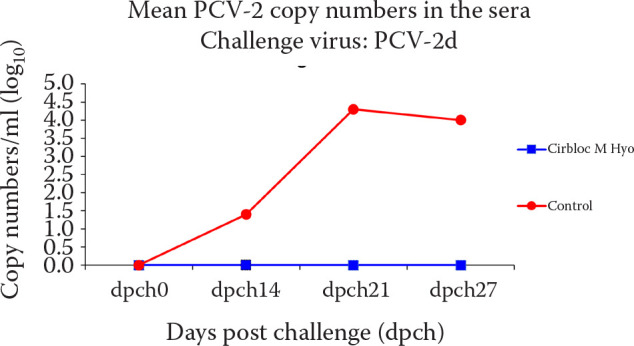
Geometric mean PCV-2d DNA copy numbers in the sera (log_10_ DNA copy number/ml)

**Figure 4:**
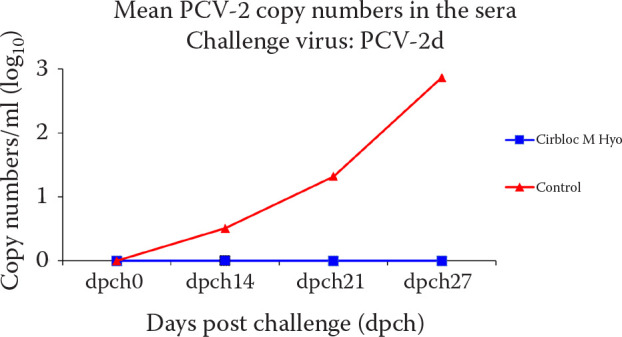
Geometric mean PCV-2d DNA copy numbers in the sera (log_10_ DNA copy number/ml) The AUC of the viraemia was significantly different between the groups (*P* = 0.000 1, *t*-test) AUC = area under the curve

The efficacy of Cirbloc M Hyo against PCV-2d infection was also confirmed at 23 weeks post-vaccination. All organ samples from vaccinated pigs showed significantly lower PCV-2 loads compared to controls (*t*-test followed by Welch test, *P =* 0.000 0). PCV-2 was barely detectable in vaccinated pigs, while high viral loads were detected in non-vaccinated control pigs across all examined organs (Table 6). When PCV-2 viraemia was measured, vaccinated pigs had PCV-2 DNA levels ranging from 0 to 0.8 log_10_/ml across different trials, with group averages never exceeding 1 log_10_/ml. In contrast, non-vaccinated control pigs exhibited high viraemia levels, reaching up to 1.5–5.0 log_10_/ml (Figures 1, 2, 3, and 4).

**Table 6:** Geometric mean PCV-2d DNA copy numbers in organ samples (log_10_ copy number/ml)

Groups	*N*	Tonsils	Mediastinal l. n.	Mesenteric l. n.	Inguinal l. n.	Lung
Cirbloc M Hyo	19	0.6	0.3	0.3	0.0	1.0
Control	17	8.0	7.6	8.0	7.2	6.9
Statistical significance		*P* = 0.000 0	*P* = 0.000 0	*P* = 0.000 0	*P* = 0.000 0	*P* = 0.000 0

l. n. = lymph nodes

## DISCUSSION

Early and long-lasting protection against *M. hyo* infection induced by the bivalent PCV-2/*M. hyo* vaccine Cirbloc M Hyo was demonstrated in this study. Three weeks post-vaccination, pigs were already protected against experimental challenge, as confirmed by a significant reduction in lung lesions observed 4 weeks post-infection.

The duration of protection was demonstrated at 23 weeks after vaccination, also assessed 4 weeks post-infection. This protection level is at the upper end of the range compared to some previously published results with other licensed PCV-2/*M. hyo* vaccines, which reported durations between 21 and 23 weeks (Witvliet et al. 2015; Mancera Gracia et al. 2021).

Regarding PCV-2, protection was evident as early as two weeks post-vaccination, representing the shortest interval reported compared to existing data (Witvliet et al. 2015; Puig et al. 2022), and as late as 23 weeks post-vaccination, which is the longest duration reported relative to previously published findings (Witvliet et al. 2015; Montane Giralt et al. 2021).

Most commercially available PCV-2 vaccines currently on the market contain the PCV-2a antigen. Given the emergence and dominance of different, more prevalent genotypes, cross-protection is critical for ensuring effective clinical protection in real-world field conditions. Many experimental studies evaluating the efficacy of PCV-2/*M. hyo* RTM or RTU vaccines have used homologous PCV-2 genotypes for the challenge (Ahn et al. 2021; Bandrick et al. 2022; Ham et al. 2023). However, cross-protection against heterologous PCV-2 genotypes has also been demonstrated in other experimental challenge studies. For instance, commercially available PCV-2a or PCV-2a/b vaccines have shown cross-protection against PCV-2d (Suh et al. 2022) or PCV-2b (Park et al. 2013; Huang et al. 2016; Sibila et al. 2020). Some authors have demonstrated protection against two different genotypes, PCV-2a and PCV-2b (Oh et al. 2021), while one study used three different genotypes – PCV-2a, PCV-2b, and PCV-2d – as challenge material (Park et al. 2019).

The ability of this novel PCV-2d and *M. hyo* RTU vaccine to confer cross-protection against all three major PCV-2 genotypes – PCV-2a, PCV-2b, and PCV-2d – was demonstrated in the present study. To our knowledge, this is the first experimental challenge study to demonstrate such broad protection using a PCV-2d-based combined PCV-2/ *M. hyo* vaccine.

PCV-2 has been recognised as a significant pathogen in PRDC. PCV-2 antigens have been detected in the cytoplasm of pulmonary macrophages, the bronchiolar epithelium, and endothelial cells, indicating that PCV-2 may exacerbate PRDC. It is often identified as a causative agent of lung disease (Kim et al. 2003; Pineyro et al. 2020). These findings suggest that PCV-2 is highly prevalent in pigs with PRDC and should be regarded as a major respiratory pathogen. The use of PCV-2 vaccination in PRDC-affected herds significantly reduces the incidence of respiratory disease and pulmonary co-infections (Fachinger et al. 2008). *M. hyo* is the primary infectious cause of EP and the most important bacterial agent contributing to PRDC (Maes et al. 2018). Pigs co-infected with PCV-2 and *M. hyo* exhibit more severe clinical symptoms, reduced weight gain, higher lung lesion scores, prolonged PCV-2 viremia, and elevated PCV-2 loads in both blood and tissues compared to pigs with mono-infections. Peribronchial lymphoid hyperplasia caused by *M. hyo* appears to serve as a key site for PCV-2 replication in the lungs (Opriessnig et al. 2004).

The availability of a new vaccine based on the most prevalent genotype of PCV-2 and an already time-proven *M. hyo* bacterin – providing confirmed cross-protection against all major genotypes, and high *M. hyo* protection with the widest known range between onset and duration of protection – will help swine producers and veterinarians better control these critical pathogens, both of which play a central role in porcine respiratory disease complex development.
